# An unusual presentation of *de novo RAC3* variation in prenatal diagnosis

**DOI:** 10.1007/s00381-024-06285-z

**Published:** 2024-01-12

**Authors:** Colombine Meunier, Marie Cassart, Karole Kostyla, Nicolas Simonis, Olivier Monestier, Aude Tessier

**Affiliations:** 1 Institut de Pathologie et de Génétique, IPG, 25, Avenue Georges Lemaitre, 6041 Gosselies, Belgium; 2 Hôpitaux Iris Sud and CHU Saint-Pierre, Brussels, Belgium; 3 Centre Hospitalier Tivoli, La Louvière, Belgium

**Keywords:** *RAC3*, Prenatal diagnosis, Cerebral malformations, Ventriculomegaly, NEDBAF

## Abstract

Pathogenic variants in *RAC3* cause a neurodevelopmental disorder with brain malformations and craniofacial dysmorphism, called NEDBAF. This gene encodes a small GTPase, which plays a critical role in neurogenesis and neuronal migration. We report a 31 weeks of gestation fetus with triventricular dilatation, and temporal and perisylvian polymicrogyria, without cerebellar, brainstem, or callosal anomalies. Trio whole exome sequencing identified a *RAC3* (NM_005052.3, GRCh38) probably pathogenic *de novo* variant c.276 T>A p.(Asn92Lys). Eighteen patients harboring 13 different and essentially *de novo* missense *RAC3* variants were previously reported. All the patients presented with corpus callosum malformations. Gyration disorders, ventriculomegaly (VM), and brainstem and cerebellar malformations have frequently been described. The only previous prenatal case associated with *RAC3* variant presented with complex brain malformations, mainly consisting of midline and posterior fossa anomalies. We report the second prenatal case of NEDBAF presenting an undescribed pattern of cerebral anomalies, including VM and polymicrogyria, without callosal, cerebellar, or brainstem malformations. All neuroimaging data were reviewed to clarify the spectrum of cerebral malformations.

*De novo* pathogenic variants of *RAC3* cause neurodevelopmental disorder with structural brain anomalies and dysmorphic facies (NEDBAF) (OMIM #618577) [1], whereas some *de novo* missense *RAC1* variants with dominant-negative effect have been associated with cerebral malformations and highly variable head circumference (OMIM #617751) [2].

*RAC3* is expressed in the central nervous system, whereas *RAC1* is expressed ubiquitously. *RAC3* belongs, like *RAC1*, to the Rho family of guanosine triphosphatases (GTPases), which are key regulators of cytoskeletal dynamics and intracellular signalling. *RAC3* is involved in neuronal migration, and both *RAC1* and *RAC3* are involved in neurogenesis. These proteins share a highly conserved G domain that mediates GDP/GTP binding and GTP hydrolysis. This region includes two regions, Switch-I and Switch-II, where most *RAC1* and *RAC3* pathogenic variants are located [3]. Hinde et al. demonstrated that dimeric *RAC1* is inactive and selectively exported to the cytoplasm, whereas monomeric *RAC1* is active and retained in the nucleus [4].

Here, we report a fetus from an unrelated and healthy Caucasian couple. Ultrasonography performed at 28 weeks of gestation (WG) demonstrated triventricular dilatation with square frontal horns, frontal lobes hypoplasia, insufficient Sylvian operculization and bilateral clubfeet (Fig. 1A).

**Fig. 1:**
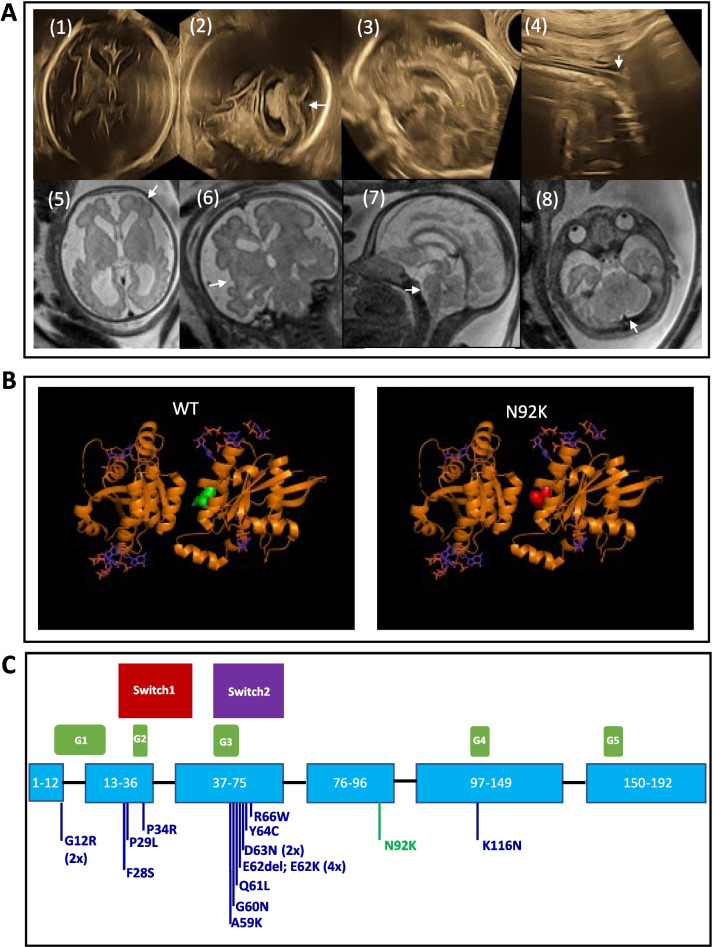
**A** Sonographic and MRI examinations performed respectively at 28 and 31 WG showed ventriculomegaly and square shape of the frontal horns with enlarged pericerebral spaces and hypoplastic frontal lobes (arrow) (1,5), abnormal Sylvian operculization (2,6: arrows), the normal corpus callosum (3 between crosses, 7) and the normal aspect of the brainstem and cerebellum (4,7,8 arrows). **B** The 3D structures of wild-type (left side) and variant (right side) *RAC3* proteins were predicted with the PyMOL application (2C2H Crystal structure of *RAC3* in complex with GDP from the Protein Data Bank: https://www.rcsb.org/). The wild-type (WT) and variant amino acids are represented in green and red respectively. In silico protein modeling indicates that the variant could interfere with *RAC3* dimer formation, because of its position at the interface between both *RAC3* proteins. Cytoplasmic accumulation of active monomeric forms of *RAC3* could be suggested, as previously described with *RAC1* variants [4]. **C** Schematic overview of the canonical *RAC3* transcript. Coding exons of *RAC3* are illustrated by light blue boxes (ENST00000306897.8), and introns by black lines. Residues 1–192 were indicated within the corresponding exons. The G boxes are represented by the green boxes. Switch I region (red) extends from residues 25–39 and Switch II region (purple) from residues 57–75. The variant in *RAC3* identified in this study is indicated by the green line, and the eighteen different variants in *RAC3* reported in previous publications are indicated using dark blue lines. Exon lengths, variants/Switch domains/G boxes positions are represented approximately to scale, but intron lengths are not

White matter thinning, expanded subarachnoid spaces, and temporal and perisylvian polymicrogyria were identified on MRI performed at 31 WG (Fig. 1A).

No cerebellar, brainstem, or callosal anomalies were observed. Parents asked for termination of the pregnancy at 32 WG. Post mortem external examination showed craniofacial dysmorphism with a large forehead, hypertelorism, downslanting palpebral fissures, bilateral epicanthus, short nose with anteverted nostrils, small mouth, thin upper lip, micro retrognathia, and short neck.

Molecular karyotype on DNA extracted from amniotic cells and blood samples from the parents was normal. Trio whole exome sequencing (WES) identified a novel *de novo* heterozygous variant c.276 T>A p.(Asn92Lys) in *RAC3* (NM_005052.3, GRCh38), absent from control databases (gnomAD 3.1), involving a highly conserved residue and predicted to be deleterious by most in silico tools (CADD score 20.5). Sequence alignment to the human reference genome, variants calling and annotation were performed using an in‐house bioinformatic pipeline. The variant is located outside the Switch domains, but in silico protein modeling suggests that it could interfere with the *RAC3* dimer formation (Fig. 1B). Considering the strong genotype/phenotype correlation, this variant was classified as probably pathogenic (Class 4).

Eighteen patients, including 16 unrelated patients and 2 half-sibs, were previously reported, harboring 13 different and essentially *de novo* missense *RAC3* variants [1, 5–9] (Table 1; Fig. 1C).

**Table 1:** Summary of cerebral malformations associated with *RAC3* pathogenic variants in the literature

	This study	Nishikawa et al. [7]	Scala et al. [8]								
			#1	#2	#3	#4	#5	#7	#8	#9	#10
*RAC3* variant	c.276 T>A p.(Asn92Lys)	c.83 T>C p.Phe28 Ser	c.187G>A p.Asp63 Asn	c.186_188 p.Glu62 del	c.191A>G p.Asp63 Asn	c.179G>A p.Gly60 Asp	c.34G>C p.Gly12 Arg	c.184G>A p.Glu62 Lys	c.348G>C p.Lys116Asn	c.187G>A p.Asp63 Asn	c.34G>C p.Gly12 Arg
Parenchyme thinning	+	+	+	+	+	+	+	+	+	-	+
VM	+	+	+	-	-	-	+	+	+	-	+
Square FH	+	+	-	-	-	-	-	-	-	-	na
CC AN	-	+	+	+	+	+	+	+	+	+	+
Midline AN	-	-	+	+	+	+	+	-	-	-	-
Gyration AN	+	-	+	+	+	+	+	+	+	+	+
Abnormal SF	+	na	na	na	na	na	na	na	na	na	na
PMG	+	-	-	-	+	+	+	-	+	+	-
Cerebellar AN	-	-	+	+	+	+	-	-	+	+	+
BS AN	-	-	+	+	-	+	-	+	-	-	+
Expanded SA spaces	+	-	+	+	+	+	+	+	+	-	-
GM heterotopia	-	-	+	-	-	-	-	-	-	-	-
Low tonsils	-	-	+	-	+	-	-	-	-	+	-

	Cabet et al. [5]	Hiraide et al. [6]	Costain et al. [1]					White et al. [9]	N = (%)		
			FI, #1	FII, #1	FIII, #1	FIV, #1	FIV, #2				
*RAC3* variant	c.196C>T p.(Arg66Trp)	c.101C>G p.Pro34 Arg	c.182A>T p.Gln61 Leu	c.86C>T p.Pro29 Leu	c.184G>A p.Glu62 Lys	c.184G>A p.Glu62 Lys	c.184G>A p.Glu62 Lys	c.176C>G p.Ala59 Gly			
Parenchyme thinning	-	+	+	+	+	+	+	-	16/19 (84)		
VM	-	+	+	+	+	-	+	-	12/19 (63)		
Square FH	-	-	-	+	na	-	+	-	4/17 (24)		
CC AN	+	+	+	+	+	+	+	+	18/19 (95)		
Midline AN	+	+	-	+	-	-	-	-	8/19 (42)		
Gyration AN	+	+	+	+	+	+	+	-	17/19 (89)		
Abnormal SF	+	na	na	na	na	na	na	na	2/2 (100)		
PMG	-	-	+	+	+	-	-	-	9/19 (47)		
Cerebellar AN	+	+	-	-	-	-	-	-	9/19 (47)		
BS AN	+	+	+	-	-	+	+	-	10/19 (53)		
Expanded SA spaces	-	-	-	-	-	-	-	-	8/19 (42)		
GM heterotopia	+	na	na	na	na	+	+	-	4/15 (27)		
Low tonsils	-	-	-	-	-	+	+	-	5/19 (26)		

Reference sequence is NM_005052.3, GRCh38

*AN* anomaly, *BS* brainstem, *CC* corpus callosum, *F* family, *FH* frontal horn, *GM* gray matter, *na* non-available information, *PMG* polymicrogyria, *SA* subarachnoid, *SF* Sylvian fissure, *VM* ventriculomegaly

Only one prenatal case has been previously reported [5]. The hallmark malformation concerned the corpus callosum (CC): thick genu, splenium thinning, and partial or total agenesis. Here, we report the first case without callosal abnormalities, suggesting that an apparently normal CC does not exclude the involvement of *RAC3*. Gyration disorders, including polymicrogyria at various locations, are the second most commonly reported neuroimaging characteristic. Cerebral parenchymal thinning with subsequent enlarged subarachnoid spaces is also often described, in contrast to a normal head circumference. Ventriculomegaly (VM) was present in more than half of cases. Until now, square frontal horns have not been reported to be associated with *RAC3*-related neurodevelopmental disorders. However, we noticed square frontal horns in previously reported cases after reviewing the existing neuroimaging data (Table 1). Structural defects of the brainstem and cerebellum are also part of the NEDBAF features with midbrain hypoplasia, elongated, flattened and/or bifid pons, abnormal cerebellar foliation, and vermis defects. Midline malformations, gray matter heterotopia, and Arnold-Chiari malformation type I have also been described.

In vitro functional studies performed on 12 variants [7, 8] have demonstrated abnormal neuronal morphology in transfected cells in comparison to the control. A gain-of-function effect with an overall increase in GDP/GTP exchange activity and reduction or suppression of GTPase hydrolytic activity. In vivo functional studies performed on five variants [7, 8] have shown severe anomalies in neuronal migration as well as defects in axon extension to the contralateral hemisphere during corticogenesis. Only two *RAC3* pathogenic variants have been previously reported outside the Switch domains [8]. There seems to be no difference in the phenotype, whether the variant is within or outside the domain.

We report here the second prenatal case of NEDBAF syndrome. The previous fetal case presented with complex brain malformations mainly consisting of midline and posterior fossa anomalies [5], whereas we identified mostly cortical malformations. Both fetuses had a fetal akinesia sequence and similar craniofacial dysmorphic features. The variation between both fetuses could be explained, at least in part, by the different localization of the variants.

Many of the brain malformations described in the NEDBAF condition are prenatal cerebral features suggestive of tubulinopathies. Cabet et al. [10] described two distinct prenatal imaging patterns related to tubulinopathies: a severe form characterized by enlarged germinal matrices, microlissencephaly, and kinked brainstem, and a mild form associated with asymmetric brainstem, corpus callosal dysgenesis, lack of Sylvian fissure operculization, and distortion of the anterior part of the interhemispheric fissure, in the absence of additional extracerebral anomalies. In the largest series of 19 fetuses with prenatal diagnosis of tubulinopathy, Brar et al. [11] reported VM, cerebellar hypoplasia, brainstem kinking, abnormalities of the CC, flat appearing Sylvian fissure, microlissencephaly, microcephaly and absence of cavum septum pellucidum. Square-shaped anterior horns described in some cases of NEDBAF are also compatible with tubulinopathies [10]. Cerebral malformations are highly variable in both conditions, and can affect the supratentorial and infratentorial parts of the brain.

In this study, we report a second prenatal case of NEDBAF presenting an undescribed pattern of cerebral anomalies, including VM with square-shaped anterior horns, and temporal and perisylvian polymicrogyria, without callosal, cerebellar, or brainstem malformations. NEDBAF should be considered as a differential diagnosis of tubulinopathies because of the similarity of cerebral malformations. Enlarged germinal matrices, microlissencephaly or microcephaly should lead to suspicion of tubulinopathy in the first place. On the other hand, extracerebral features, such as arthrogryposis, should point towards *RAC3-*fetopathy. Furthermore, we outline that various brain malformations can lead to the same diagnosis, highlighting the benefit of fast whole exome sequencing in prenatal cases.

## Data Availability

No datasets were generated or analysed during the current study.
